# Prospective, multicenter French study evaluating the clinical impact of the Breast Cancer Intrinsic Subtype-Prosigna^®^ Test in the management of early-stage breast cancers

**DOI:** 10.1371/journal.pone.0185753

**Published:** 2017-10-18

**Authors:** Delphine Hequet, Céline Callens, David Gentien, Benoit Albaud, Marie-Ange Mouret-Reynier, Coraline Dubot, Paul Cottu, Cyrille Huchon, Sonia Zilberman, Helene Berseneff, Cyril Foa, Rémy Salmon, Aurélie Roulot, Florence Lerebours, Anne Salomon, Nadeem Ghali, Pascale Morel, Qianyi Li, Anne Cayre, Jean-Marc Guinebretière, John Hornberger, Frédérique Penault-Llorca, Roman Rouzier

**Affiliations:** 1 Department of Surgical Oncology, Institut Curie-Centre René Huguenin, St Cloud, France; 2 INSERM-U900 –Bioinformatics, biostatistics, epidemiology and computational systems. Biology of cancer, St Cloud, France; 3 Department of Pharmacogenomics, Institut Curie, Paris, France; 4 Institut Curie, PSL Research University, Translational Research Department, Genomic platform, Paris, France; 5 Department of Medical Oncology, Centre Jean Perrin, Clermont-Ferrand, France; 6 Department of Medical Oncology, Institut Curie-Centre René Huguenin, St Cloud, France; 7 Department of Gynecology, Poissy-St Germain hospital, Poissy, France; 8 Department of Gynecology, Tenon Hospital, Paris, France; 9 Department of Gynecology, René Dubos Hospital, Pontoise, France; 10 Department of Medical Oncology, Private Hospital Clairval, Marseille, France; 11 Department of Surgery, Private Hospital Les Peupliers, Paris, France; 12 Department of Pathology, Institut Curie-Centre René Huguenin, St Cloud, France; 13 NanoString Technologies, Inc., Seattle, WA, United States of America; 14 Cedar Associates LLC, Menlo Park, CA, United States of America; 15 Department of Pathology, Centre Jean Perrin-UMR INSERM 1240, University Clermont Auvergne, Clermont-Ferrand, France; 16 Stanford University School of Medicine, Department of Internal Medicine, Stanford, CA, United States of America; Universita Campus Bio-Medico di Roma, ITALY

## Abstract

**Purpose:**

The Prosigna^®^ breast cancer prognostic gene signature assay identifies a gene-expression profile that permits the classification of tumors into subtypes and gives a score for the risk of recurrence (ROR) at 10 years. The primary objective of this multicenter study was to evaluate the impact of Prosigna’s assay information on physicians’ adjuvant treatment decisions in patients with early-stage breast cancer. Secondary objectives were to assess confidence of practitioners in their therapeutic recommendations before and after the added information provided by the Prosigna assay; and to evaluate the emotional state of patients before and after the Prosigna test results.

**Methods:**

Consecutive patients with invasive early-stage breast cancer were enrolled in a prospective, observational, multicenter study carried out in 8 hospitals in France. The Prosigna test was carried out on surgical specimens using the nCounter^®^ Analysis System located at the Institut Curie. Both before and after receiving the Prosigna test results, physicians completed treatment confidence questionnaires and patients completed questionnaires concerning their state of anxiety, the difficulties felt in face of the therapy and quality of life. Information was also collected at 6 months regarding the physicians’ opinion on the test results and the patients’ degree of anxiety, difficulties with therapy and quality of life.

**Results:**

Between March 2015 and January 2016, 8 study centers in France consecutively enrolled 210 postmenopausal women with estrogen receptor (ER) positive, human epidermal growth hormone-2 (HER-2) negative, and node negative tumors, either stage 1 or stage 2. Intrinsic tumor subtypes as assessed by the Prosigna test were 114 (58.2%) Luminal A, 79 (40.3%) Luminal B, 1 (0.5%) HER-2 enriched (HER-2E), and 2 (1.0%) basal-like. Before receiving the Prosigna test results, physicians categorized tumor subtypes based on immunohistochemistry (IHC) as Luminal A in 126 (64%) patients and Luminal B in 70 (36%) patients, an overall discordance rate of 25%. The availability of Prosigna assay results was significantly associated with the likelihood of change in treatment recommendations, with 34 patients (18%) having their treatment plan changed from Adjuvant Chemotherapy to No Adjuvant Chemotherapy or vice versa (p<0.001, Fisher’s exact test). Prosigna test results also decreased patients’ anxiety about the chosen adjuvant therapy, and improved emotional well-being and measures of personal perceptions of uncertainty.

**Conclusions:**

The results of this prospective decision impact study are consistent with 2 previous, identically designed studies carried out in Spain and Germany. The availability of Prosigna test results increased the confidence of treating physicians in their adjuvant treatment decisions, and led to an 18% change in chemotherapy treatment plan (from Adjuvant Chemotherapy to No Adjuvant Chemotherapy or vice versa). Prosigna testing decreased anxiety and improved measures of health-related quality of life in patients facing adjuvant therapy. The 25% discordance between Prosigna test and IHC subtyping underlines the importance of molecular testing for optimal systemic therapy indications in early breast cancer.

## Introduction

Molecular biomarkers play an increasingly important role in helping define prognosis and predict response to specific treatments for patients with cancer. Molecular diagnostic tests are now routinely used in the clinic to tailor therapy to the individual characteristics of a tumor. Early-stage breast tumors are clinically and genomically heterogeneous, enabling their classification into subtypes that permit optimization of treatment based on prognosis [1]-[3].

Several validated molecular subtyping tests for breast cancer based on gene expression profiling are now routinely used in clinical practice. In addition to the molecular tests that use DNA microarray analysis, the pioneering work of Sørlie et al has led to a classification of breast cancer by distinct biologic subtypes, or intrinsic subtypes [4]. By analyzing surgical specimens of human breast tumors using complementary DNA microarrays, this group identified variations in gene expression patterns that yielded a distinctive “molecular portrait” of breast cancer, according to which tumors could be classified into 5 intrinsic subtypes with distinct clinical outcomes: Luminal A, Luminal B, human epidermal growth hormone 2 (HER2) over-expression, basal, and a normal-like group [2], [3]. Based on this classification, a risk model was developed using a 50-gene subtype predictor (PAM50). The Prosigna test based on the PAM50 gene signature measures the expression of 50 genes in a surgically resected breast cancer sample to classify a tumor as one of 4 intrinsic subtypes (Luminal A, Luminal B, HER2-enriched [HER-2E], and basal-like). The assay uses the 50-gene expression profile, weighted together with clinical variables, to generate a risk category and numerical score. The score is reported on a 0-100 scale (risk of recurrence [ROR] score), which is correlated with the probability of distant recurrence at 10 years for post-menopausal women with hormone receptor-positive, early stage breast cancer not receiving chemotherapy. The test is FDA 510(k) cleared in the U.S and Conformité Européene (CE)-marked in Europe for use on FFPE tissue. The utility of the Prosigna test in making prognosis and treatment decisions has been clinically validated [5], [6] and it is included as an option for risk assessment in several clinical practice guidelines such as those of the St. Gallen International Breast Cancer Conference [7], European Society for Medical Oncology (ESMO) [8], and American Society of Clinical Oncology (ASCO) [9].

Even though a biomarker assay may be clinically validated, it remains important to learn how physicians respond to the information provided, and to estimate the magnitude of change in clinical recommendations that occurs before and after knowledge of the assay [9–13]. Effect on clinical recommendations and management is a part of what several guidelines refer to as the clinical utility of the test [9], [14], [15]. Based on prior studies of the Prosigna test, we predicted that physicians would recommend a switch to chemotherapy if the Prosigna test result was reported as high risk and the patient previously was recommended to have hormonal therapy only. By contrast, we predicted that physicians would recommend a switch to hormonal therapy only if the Prosigna result was reported as low risk and the patient previously was recommended to have chemotherapy. We also sought to gain more experience with the concordance of Prosigna test results when performed in local clinic settings or in a centralized laboratory.

The present study is identical in design to 2 previous studies that have been completed. The first was carried out in 15 hospitals across Spain affiliated with the Spanish breast cancer group El Grupo Español de Investigación en Cáncer de Mama (GEICAM) [16], and the second at 11 breast centers across Germany by the West German Study Group [17]. The earliest such decision impact studies were carried out using the Oncotype DX^®^ assay [18].

The primary objective of the study was to evaluate the impact of the result of the Prosigna test on changes in risk assessment and in recommendations of adjuvant therapy. Secondary objectives were to evaluate practitioners’ confidence in therapeutic indications before and after the Prosigna test results and to evaluate selected measures of health-related quality of life of patients.

## Patients and methods

This prospective, observational study was coordinated by the Institut Curie with 7 additional study sites (total of 8). The Comité de Protection des Personnes de Paris V (Paris V Institutional Review Board), specifically approved this study on 02 May 2014, before the study start. A total of 210 patients were enrolled at 8 sites in France between March 2015 and January 2016. Eligible patients were postmenopausal women with invasive early-stage breast cancer (T1-T2), nodal status N0, pN0 (i+), or pN0 (mol+), who had no contraindication for adjuvant chemotherapy. Eligibility criteria also included an Eastern Cooperative Oncology Group (ECOG) score of 0 or 1, ability to complete the questionnaires without assistance, and provision of written informed consent. Patients were excluded if they had estrogen receptor (ER) negative tumors or tumors overexpressing HER2, or had metastatic disease. Estrogen receptor status was determined by immunohistochemistry (IHC). HER2 status was determined by IHC and/or fluorescence in situ hybridization (FISH).

The trial was registered with ClinTrials.gov (NCT02395575) on 04 March 2015. The authors confirm that all ongoing and related trials for this drug/intervention are registered.

### Tumor sample assessments

The anatomic pathology laboratory at each center was trained on the study and responsible for preparation of the specimen and delivery to the Institut Curie. Prosigna testing was carried out according to manufacturer specifications (Prosigna assay package insert) using the nCounter Analysis System at the Institut Curie. Test results (ROR score, risk of recurrence, risk group, and molecular subtype) were provided back to the center. There was a 7-day maximum turnaround time to deliver the Prosigna report to the clinician for discussion with the patient, with 2 runs a week. Prosigna test results classified tumors according to intrinsic subtypes (Luminal A, Luminal B, HER2-enriched, basal-like) and ROR risk groups (low risk, 0-40; intermediate risk, 41-60; and high risk, 61-100).

A second set of FFPE tumor sections was subsequently analyzed with the Prosigna test in an independent replication laboratory (Centre Jean Perrin—Clermont Ferrand) to assess concordance with the central laboratory. ER and PR were analyzed by IHC, HER2 status was analyzed by IHC and confirmed by FISH when indicated (when positive); and Ki67 was assessed by IHC. IHC was performed locally, and subtypes based on IHC results were assessed by the treating physician. A threshold of 10% was used to define positivity for ER and PR. Prosigna test-based subtypes were compared to the physician’s assessment based on local IHC results, using the St. Gallen 2013 criteria [14].

### Physician questionnaires

After obtaining patient consent, prior to the Prosigna test, the physician completed a questionnaire with information on the patient’s disease characteristics and the adjuvant therapy planned. The same questionnaire has been used in previous studies [16], [17]. The initial treatment recommendation was determined in a multidisciplinary meeting and was based on standard clinical and pathological factors and the IHC results. Physicians also recorded their confidence in this recommendation. Upon receipt of the Prosigna test results (ROR and intrinsic tumor subtype), physicians again in a multidisciplinary team provided information regarding the intended adjuvant therapy and their confidence in that decision. At a 6-month follow-up visit, physicians completed a final questionnaire concerning their opinion on the usefulness of the test, their confidence in the test results, and the patients' medical follow-up elements.

### Patient questionnaires

At the inclusion visit (signing of the informed consent form), patients completed pre-Prosigna test questionnaires regarding their state of anxiety, difficulties they felt facing the therapy, and quality of life. Once the test results were obtained, the investigator and the patient together completed the post-Prosigna test questionnaire assessing anxiety and other difficulties felt facing therapy. At the 6-month follow-up visit, patients again provided information regarding their degree of anxiety, possible difficulties felt facing the therapy, and quality of life. Anxiety was assessed using the State-Trait Anxiety Inventory (STAI), a 40-item questionnaire designed to measure 2 aspects of anxiety: anxiety state (situational, circumstantial anxiety); and anxiety trait (stable personality traits that predispose to anxiety) [19]. Health-related quality of life was assessed using the Functional Assessment of Cancer Therapy–General, version 4 (FACT-G v.4), which evaluates 4 domains in patients undergoing cancer therapy: physical, social/family, emotional, and functional [20]. The Decisional Conflict Scale (DCS) was used to assess patients’ perceived level of decisional conflict [21].

### Statistical analyses

The sample size of 200 patients was calculated to provide a one-sided 95% lower-limit confidence interval, with a 0.05 distance from the sample proportion (0.25) to the lower limit (Clopper–Pearson). Clinical and demographic characteristics were described by mean, median, standard deviation, range and frequency. All questionnaires were analyzed and results compared (total scores and the component scores) among all the patients, as well as for subgroups by Prosigna test risk category. The equality of the means of continuous variables stratified by ROR group status was assessed by one-way analysis of variance [22]. Concordance between physician judgement and Prosigna test results was assessed with the kappa statistics. (A commonly used guide to interpreting kappa statistics defines a kappa of less than 0 as poor concordance; kappa of 0.01 to 0.20 as slight agreement; kappa of 0.21 to 0.40 as fair agreement; kappa of 0.41 to 0.60 as moderate agreement; kappa of 0.61 to 0.80 as substantial agreement; and kappa of 0.81 to 0.99 as almost perfect agreement [23]). The proportion of patients whose choice of therapy changed after the Prosigna test results was calculated for the sample population as a whole, and for key subgroups. The association between the proportions was tested with Fisher’s exact test for (row ≥ 2) x (column ≥ 2) contingency tables [24]. Changes in practitioners’ confidence in the planned therapies before and after the test results was analyzed by calculating the number and percentage responding positively that they are “confident in intended treatment (optimal for patient)”; and physicians’ perceptions of the Prosigna test were similarly evaluated with questions asking whether Prosigna test results “provided additional clinically useful information”, “influenced treatment recommendations”, and whether they “would use Prosigna [test] again”. The differences between pre- and post-Prosigna were assessed with Student’s t test [25], [26]. All tests were 2-sided at 0.05 level of statistical significance to reject the null hypothesis (e.g., equality of means). Statistical analysis was performed in STATA^®^ 15.0 (StataCorp, LLC, College Station, Tx).

## Results

### Patient and tumor characteristics, and Prosigna test results

**Fig 1:**
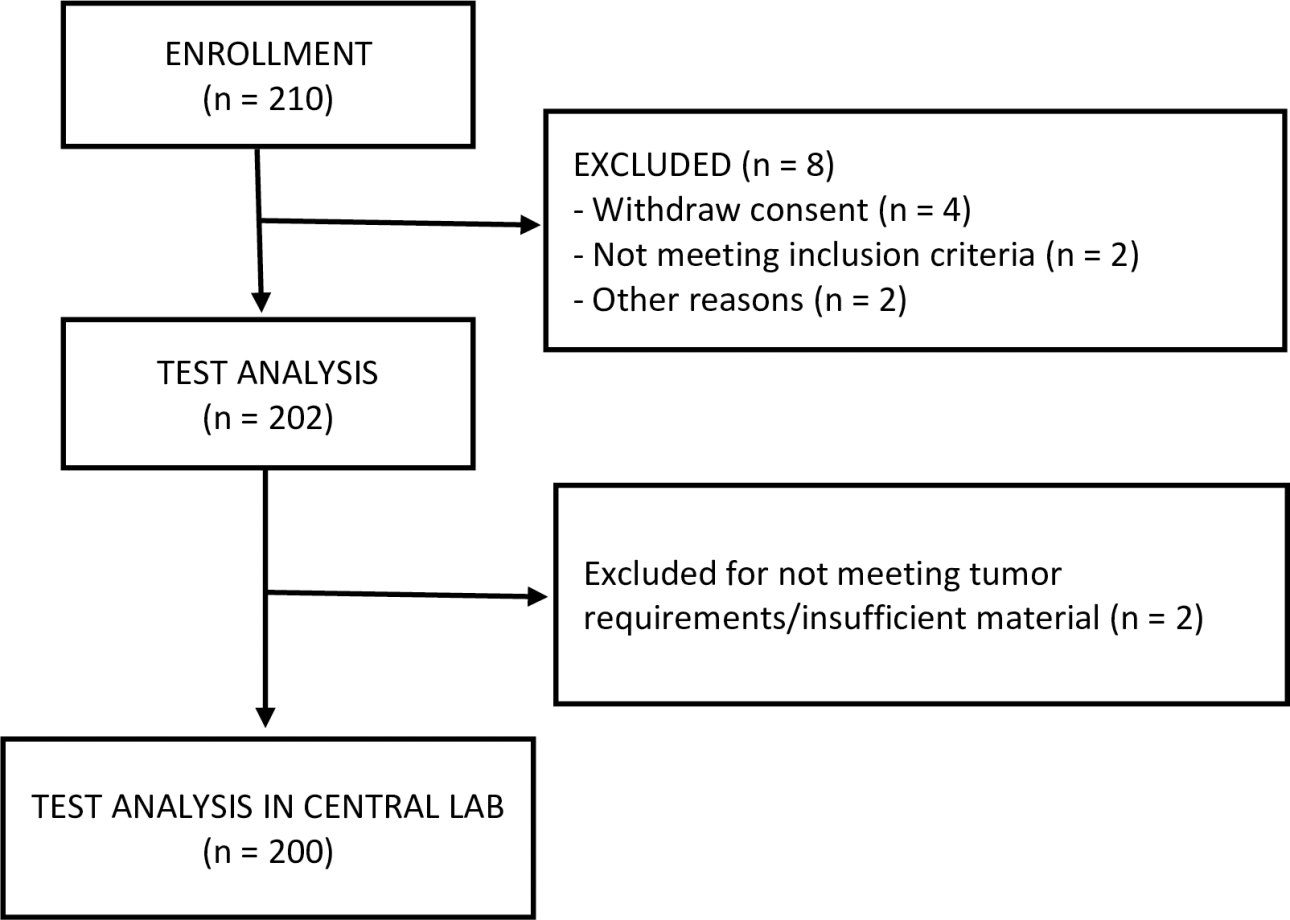
Patient flow diagram.

Two hundred and ten patients were enrolled at 8 sites in France. Eight of these patients were excluded (4 withdrew consent, 2 were determined to be ineligible, and 2 were excluded for other reasons (Fig 1). The remaining 202 samples were reviewed for suitability in the local pathology laboratory. Of these, 2 were excluded for failing to meet tumor requirements or having insufficient material. The Prosigna test was successfully completed for 200 patients at the central laboratory and 181 patients at the replication laboratory (too little material remained for analysis by the replication laboratory in 19 cases). Patient characteristics by ROR group are shown in Table 1. Of the 200 patients enrolled, 198 (99%) were at least 50 years of age (the mean age was 61.9 years). Most (158 [79%]) had T1 tumors; 42 (21%) had T2 tumors (the percentage of patients with T1 tumors was highest in the low ROR group [85%], and the percentage of patients with T2 tumors was highest in the high ROR group [43%]). A total of 172 (86%) patients were PR positive (with a similar proportion positive in each ROR group). Approximately half had Ki67 >14% (the percentage of patients with Ki67 >14% increased with higher ROR).

**Table 1:** Patient and tumor characteristics.

Characteristic	Type	All		ROR low		ROR intermediate		ROR high		p-value*
		N	%	n	%	n	%	n	%	
Age (years)	<50	2	1%	1	1%	1	1%	0	0%	0.75
	> = 50	198	99%	92	99%	66	99%	40	100%	
Tumor size	T1	158	79%	79	85%	56	84%	23	58%	0.001
	T2	42	21%	14	15%	11	16%	17	43%	
PR	Positive	172	86%	84	90%	54	81%	34	85%	0.21
	Negative	28	14%	9	10%	13	19%	6	15%	
Ki67	<14%	97	49%	70	75%	21	31%	6	15%	<0.0001
	> = 14%	98	49%	20	22%	45	67%	33	83%	
	Unknown	5	3%	3	3%	1	1%	1	3%	
TOTAL		200		93		67		40		

* Based on one-way ANOVA

A total of 196 patients had all 3 intrinsic tumor subtype assessments (using IHC by the physician according to St. Gallen 2013 criteria both before and after availability of Prosigna test results; and Prosigna assay done at the central laboratory). Before receiving the Prosigna test results, physicians categorized subtypes by St Gallen IHC criteria as Luminal A in 126 (64%) patients and Luminal B in 70 (36%) patients. Post-Prosigna assay, these values were 111 (56.6%) Luminal A, 84 (42.9%) Luminal B, and 1 (0.5%) HER2-E. Tumor subtypes as assessed by the Prosigna test were 114 (58.2%) Luminal A, 79 (40.3%) Luminal B, 1 (0.5%) HER-2E, and 2 (1.0%) basal-like. The average percentage of Ki67 in Luminal A and Luminal B samples is shown in S1 Fig. A receiver operating characteristic (ROC) curve to predict the optimal threshold for discrimination of Luminal A and Luminal B is shown in S2 Fig.

There was 72% concordance at the individual level between tumor subtype classification by IHC and by the Prosigna gene signature assay results (kappa = 0.43) (Table 2, S3 Fig). Concordance between the Prosigna assay results and physicians’ post-Prosigna test assessment was 95% (kappa = 0.91).

**Table 2:** Intrinsic tumor subtype results by physician judgement and Prosigna test results.

		Subtype by Prosigna test results				
		Lum A	Lum B	HER2-E	Basal	TOTAL
Subtype by IHC	Lum A	94	31	1	0	126
	Lum B	20	48	0	2	70
	HER2-E	0	0	0	0	0
	Basal	0	0	0	0	0
	TOTAL	114	79	1	2	196
Concordance		75%	69%	NA	NA	72%

### Risk of recurrence (ROR)

Physicians assessed the risk of 10-year distant recurrence for 194 patients with available data as “low” for 125 (64.4%) patients, “intermediate” for 62 (32.0%) patients, and “high” for 7 (3.6%) patients. The Prosigna assay assessed ROR as “low” for 88 (45.4%) patients, “intermediate” for 66 (34.0%) patients, and “high” for 40 (20.6%) patients (kappa = 0.29) (Table 3). No Luminal B tumors were classified in the ROR low-risk group, and no Luminal A tumors were classified in the ROR high-risk group; the 1 tumor HER2-E tumor was classified as high ROR and the 2 basal-like tumors as intermediate ROR.

**Table 3:** Concordance between pre-test physician assessment and Prosigna test results in risk of 10-year distant recurrence.

		Risk according to physician			
		Low	Intermediate	High	TOTAL
Risk according to Prosigna test	Low	78	9	1	88
	Intermediate	33	31	2	66
	High	14	22	4	40
	TOTAL	125	62	7	194

Concordance between physician assessment and Prosigna test: kappa = 0.29.

We assessed differences in terms of survival given by Prosigna and by the on-line mathematical model Predict (S4 Fig). There is a correlation between both predicted survivals. However, note that Predict provides overall survival whereas Prosigna provides disease-free survival. Moreover, mathematical models such as Predict do not take into account the value of Ki67 but consider a "positive" Ki or "negative" Ki.

### Adjuvant therapy recommendations before and after Prosigna test

The availability of Prosigna assay results was significantly associated with the likelihood of change in treatment recommendations, with 34 patients (18%) having their recommendation for adjuvant treatment changed (from No Adjuvant Chemotherapy to Adjuvant Chemotherapy or vice versa) (p<0.001, Fisher’s exact test). Table 4 shows physicians’ adjuvant treatment recommendations before and after Prosigna test results, by Prosigna test risk category. Availability of the Prosigna assay results led to a decrease in the number of recommendations for no adjuvant chemotherapy (25 patients were changed from No Adjuvant Chemotherapy to Adjuvant Chemotherapy, and 9 patients were changed from Adjuvant Chemotherapy to No Adjuvant Chemotherapy). In total there were 135 patients scheduled for no adjuvant chemotherapy before the Prosigna test results, with 119 after; and 59 patients scheduled to receive adjuvant chemotherapy before the Prosigna test results, with 75 after. The greatest change was observed for the Prosigna test high-risk patients, 38% of whom had their regimen switched (from No Adjuvant Chemotherapy to Adjuvant Chemotherapy or vice versa), versus 8% in the low-risk category and 18% in the intermediate risk category (Table 4).

**Table 4:** Adjuvant treatment recommendation pre- and post-Prosigna test results, by Prosigna test risk category.

	Low Risk	Intermediate Risk	High Risk	TOTAL	Fisher's exact test
**Switched type of regimen**	**7**	**12**	**15**	**34**	<0.001
%	8%	18%	38%	18%	
No CT to CT	0	10	15	25	
CT to no CT	7	2	0	9	

Only patients for whom both pre- and post-Prosigna test risk assessment were available were included.

### Physicians’ confidence in treatment recommendation before and after Prosigna test results

A total of 192 cases were reviewed to evaluate the medical oncologists’ confidence in their treatment decisions before and after the availability of Prosigna test results. The physicians’ confidence in the intended treatment increased with Prosigna test results in 39% of cases and decreased in 11%; 51% of physicians had no change in confidence. For physicians with 6-month data, 74% agreed or strongly agreed that Prosigna test results provided additional clinically useful information (75% at the immediate post-Prosigna test timepoint); and 76% agreed or strongly agreed that Prosigna test results influenced treatment recommendations (61% at the post-Prosigna test timepoint). When queried at 6 months, 98% of physicians agreed or strongly agreed that they would use Prosigna test again (96% immediately post-Prosigna test).

### Patient-reported outcomes

After the post-Prosigna test treatment recommendations, patients’ STAI component of state anxiety was statistically significantly decreased post-Prosigna test relative to pre-Prosigna test (p = 0.02) (S1 Table). Changes in state anxiety and social/family well-being differed significantly across the ROR categories; the Prosigna test was most helpful in decreasing state anxiety for patients with ROR low risk. Significant improvements were also seen on the DCS (p <0.001 for the “informed” and “values clarity” components as well as for the DCS overall; p = 0.008 for the “uncertainty” component). The functional assessment of emotional well-being was also significantly improved post-Prosigna relative to pre-Prosigna test results (p < 0.001). Data for the STAI and Functional Assessment are available from the 6-month evaluation, and show additional small improvements in state anxiety (mean 43.3 pre-Prosigna test, 41.5 post-Prosigna test, and 39.9 at 6 months), and emotional well-being (mean 16.8, 17.5, and 17.64, respectively) (S2 Table).

### Concordance in Prosigna test subtyping and ROR between central and replication laboratories

A total of 181 samples were analyzed by the replication laboratory. Intrinsic tumor subtypes as determined by the replication laboratory were concordant with those from the central laboratory in 94% of cases (kappa = 0.88) (3 tumors were categorized as Luminal B by the central laboratory and Luminal A by the replication laboratory). Concordance in Prosigna test ROR categories between central and replication laboratory was 91% (kappa = 0.86) (the replication laboratory reported 3 more low-risk, 2 fewer intermediate-risk, and 1 fewer high-risk tumors relative to the central laboratory).

## Discussion

While adjuvant chemotherapy is proven to provide an average survival gain for the population of postmenopausal women with ER+/HER2- breast tumors, individuals may vary in their prognosis and how much gain they will derive from adjuvant chemotherapy. The addition of adjuvant chemotherapy to endocrine therapy in these patients provides an average of <5% increase in 15-year survival. Some patients treated with adjuvant chemotherapy benefit, but some will undergo a treatment with risk of toxicities. A tumor biomarker assay is useful if it identifies a group of patients for whom the absolute benefit of adjuvant chemotherapy could not exceed 2% to 3%, which is roughly equal to the risk of serious toxicities. For example, a patient with a grade 1, ER positive, progesterone receptor-positive, HER2-negative breast cancer has a 10-year ROR of approximately 10% to 15%; adjuvant endocrine therapy would reduce this risk by approximately one-third to one-half. Assuming that adjuvant chemotherapy will further reduce her ROR by approximately one-third, a recommendation for treatment to similar patients will only benefit 2% to 3%, the same number who will be harmed by toxicities of therapy [9].

This study was designed to evaluate the influence on risk assessment and subsequent adjuvant treatment recommendations of information provided by Prosigna Gene Signature assay added to clinicopathological factors. Risk assessment and use of adjuvant therapy has been shown to vary between institutions, regions, and countries [27], [28]. One of the reasons for research in biomarkers and related risk algorithms is reduce uncertainty in risk assessments so that patients can anticipate and experience a consistent and reliable approach to initial breast cancer management regardless of the care setting, or who they see.

In the present study, 205 postmenopausal women with ER positive, HER2 negative, and node negative stage 1 or 2 tumors were enrolled at 8 centers throughout France. The study was coordinated by the Institut Curie. In this study, physicians’ determination of intrinsic tumor subtypes changed notably with the availability of the Prosigna test results, largely driven by re-classification of Luminal A tumors to Luminal B tumors. This demonstrates that IHC-based subtype classification is suboptimal and can contribute to a misestimation of a patient’s risk. In addition, recent results demonstrated no benefit of chemotherapy in Luminal A patients [29]; therefore, correct determination of tumor subtype can contribute to the decision to spare unnecessary chemotherapy. Concordance between the physicians’ post-Prosigna test determination of intrinsic tumor subtypes and the Prosigna assay results was 95%, indicating that physicians strongly agreed with the Prosigna test results after being made aware of them, and signifying that they recognize the importance of the Prosigna test results for their decision making in this setting.

Duplication of Prosigna testing at the replication laboratory demonstrated excellent reproducibility of test results, indicating that the Prosigna test is reliable when performed at local institutions without the need for a central laboratory. These “real-life” concordance results confirmed the Prosigna analytical validation study results. Very few discordances were observed, for which the clinical impact was limited, mainly resulting from tumor heterogeneity [30].

Compared to the physicians’ assessments, the Prosigna assay categorized more patients as having a high risk of 10-year distant recurrence (3.6% by the physicians, 20.6% by Prosigna test results), and fewer patients at low risk (64.4% by the physicians and 45.4% by Prosigna test results). The decision of whether to or not to offer adjuvant chemotherapy is highly dependent on the patient population and study center where the patient is seen before the genomic test is performed. Importantly, patients in this decision impact study were enrolled consecutively by the study investigators, thus eliminating the potential for physician bias in selecting only patients whose treatment is uncertain. In this study the availability of Prosigna test results led to an increase in recommendations for chemotherapy, as patients were more accurately characterized by genomic testing, ROR and molecular subtype, potentially contributing to a reduction in the rate of early recurrence.

Physicians changed their recommendations for adjuvant treatment for 18% of patients, with the proportion varying by Prosigna test risk strata. The highest proportion of changes in planned adjuvant treatment occurred in the Prosigna test high-risk group, in which 38% of patients had their regimen switched (from No Adjuvant Chemotherapy to Adjuvant Chemotherapy or vice versa). The rate of change was 8% for patients in the low-risk strata and 18% for patients in the intermediate-risk strata.

(For patients in intermediate risk groups, the decision regarding chemotherapy is essentially made based upon molecular subtype, as per Prosigna, and clinical patient characteristics).

As in previous studies, availability of the Prosigna test results increased physicians’ confidence in their treatment decisions (in 39% of cases in the present study; 42% and 89% in the Spanish and German studies, respectively), indicating the value to physicians of knowing both ROR score and molecularly based intrinsic subtype for decision making in this setting. Consistent with the physicians’ confidence in the Prosigna test results, receipt of these results caused physicians to change their adjuvant treatment plans for 18% of patients in this study (20% and 18% in the Spanish and German studies, respectively).

The availability of Prosigna test results decreased patients’ anxiety about the chosen adjuvant therapy, and improved emotional well-being and several components of the DCS, which measures personal perceptions of uncertainty in choosing options. This demonstrates that the Prosigna test results have an overall positive psychosocial impact on patients.

### Limitations

Of the 210 patients enrolled, approximately 150 to 170 provided data on measures of health-related quality of life. Missing quality of life data has been acknowledged as a challenge in cancer trials [31]. In this study, patients were enrolled sequentially, decreasing the likelihood for selection bias.

Multigene molecular tests are becoming generally accepted in medical practice to tailor adjuvant treatment and evaluate prognosis in women with ER+ breast cancer. A number of such assays are commercially available, and are supported by ample evidence and endorsement in major guidelines. Each of the assays relies upon a different set of genes, and so may yield different results. Going forward, it will be important to monitor and compare experiences using the various assays as more patients are diagnosed and new treatments are introduced.

## Conclusions

The results of this multicenter French study are consistent with previous reports of identically designed studies carried out by the GEICAM group in Spain [16], and the West German Study Group [17]. As in these 2 previous studies, the present study supported the utility of the Prosigna test in providing information that physicians value and incorporate into their clinical decision-making process. Availability of the Prosigna test results increased physician confidence in prognosis and resulting adjuvant therapy plans, and influenced treatment decisions. Physicians expressed confidence in Prosigna assay results by incorporating the results into their assessment of prognosis and adjuvant treatment decisions to a substantial degree. The additional information provided by the Prosigna test decreased patients’ anxiety and uncertainty in facing their treatment options. Concordance between Prosigna risk results and IHC-based physician assessment was low, indicating the importance of molecular testing.

## Supporting information

S1 Fig Ki67 according to Luminal A/Luminal B classification. (TIF)

S2 Fig ROC Curve to Predict Optimal Threshold for Luminal A/Luminal B. (TIF)

S3 Fig Intrinsic subtype distribution by local assessment (IHC) vs. Prosigna. (TIF)

S4 Fig Comparison of overall survival by Predict and disease-free survival by Prosigna. (TIF)

S1 Table Changes in anxiety, decisional conflict, and functional status according to ROR group. (DOCX)

S2 Table Anxiety, decisional conflict, and functional status pre- and post-Prosigna, and at 6-month follow- up. (DOCX)

S1 Appendix TREND Statement Checklist. (PDF)

S2 Appendix Trial Protocol: Prospective study evaluating the clinical impact of the Breast Cancer Intrinsic Subtype-Prosigna Test (Assay) in the management of early-stage breast cancers. dx.doi.org/10.17504/protocols.io.jrucm6w
(DOC)
