# Supplementary figures and images for: Prospective, multicenter French study evaluating the clinical impact of the Breast Cancer Intrinsic Subtype-Prosigna® Test in the management of early-stage breast cancers
Source: PLoS One. 2017 Oct 18;12(10):e0185753. doi: 10.1371/journal.pone.0185753 (PMC5646764; doi:10.1371/journal.pone.0185753)

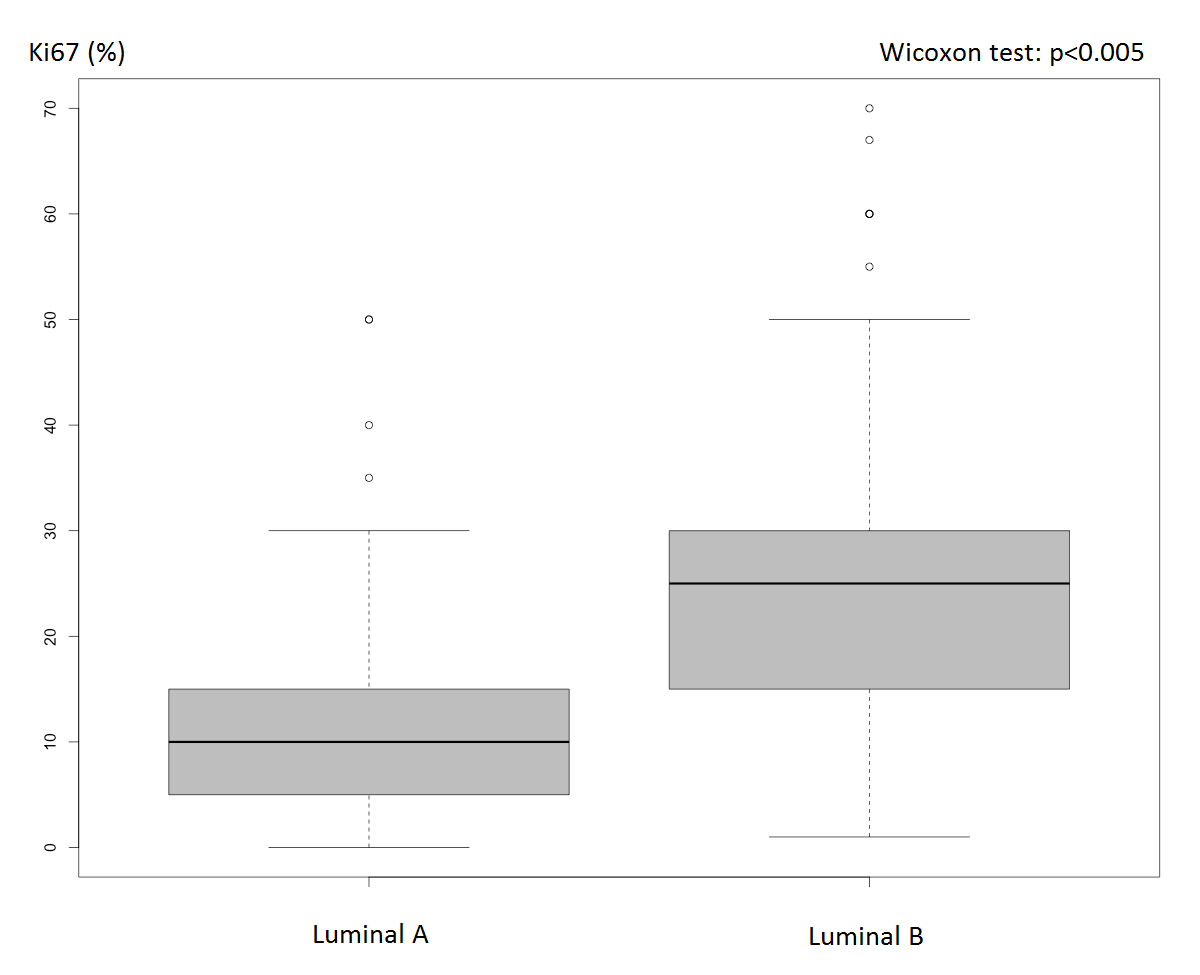

Supplement: S1 Fig — (TIF) [file pone.0185753.s001.tif]

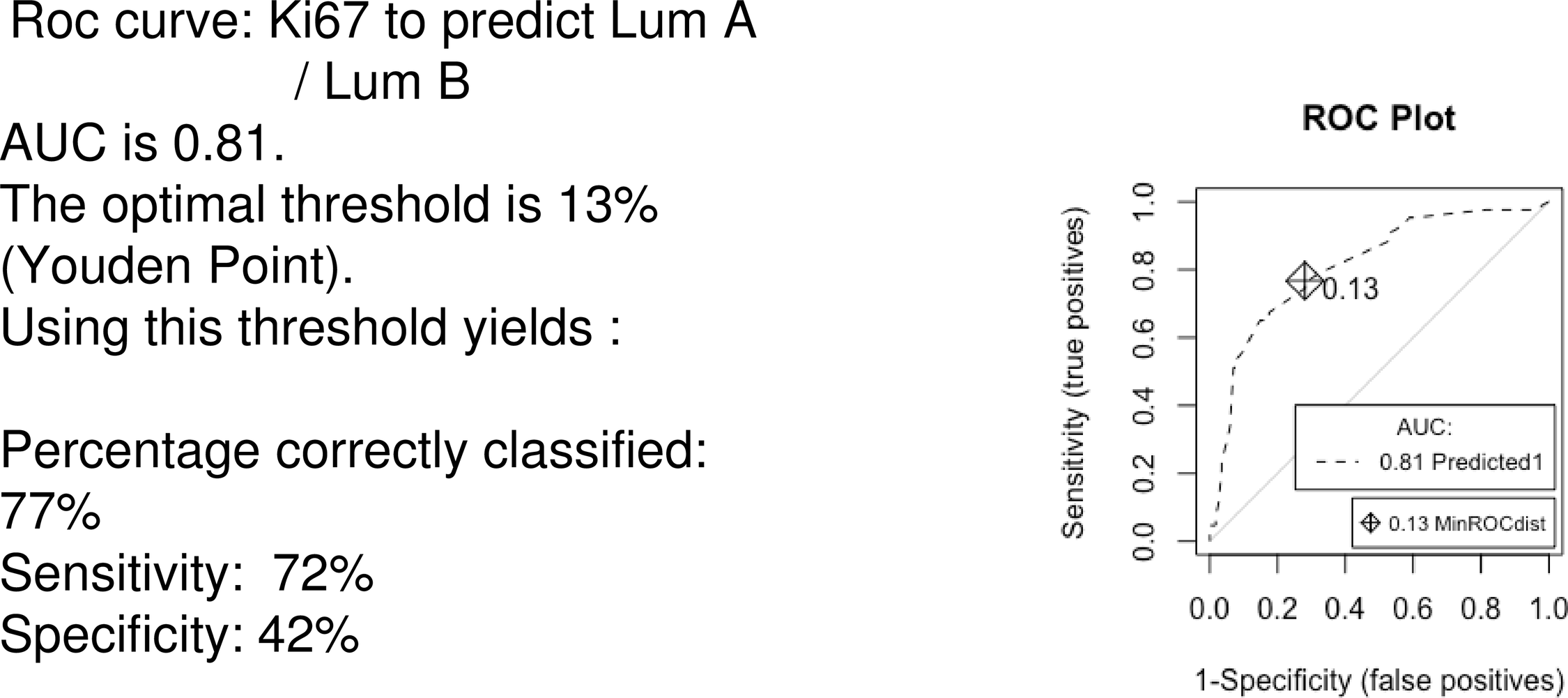

Supplement: S2 Fig — (TIF) [file pone.0185753.s002.tif]

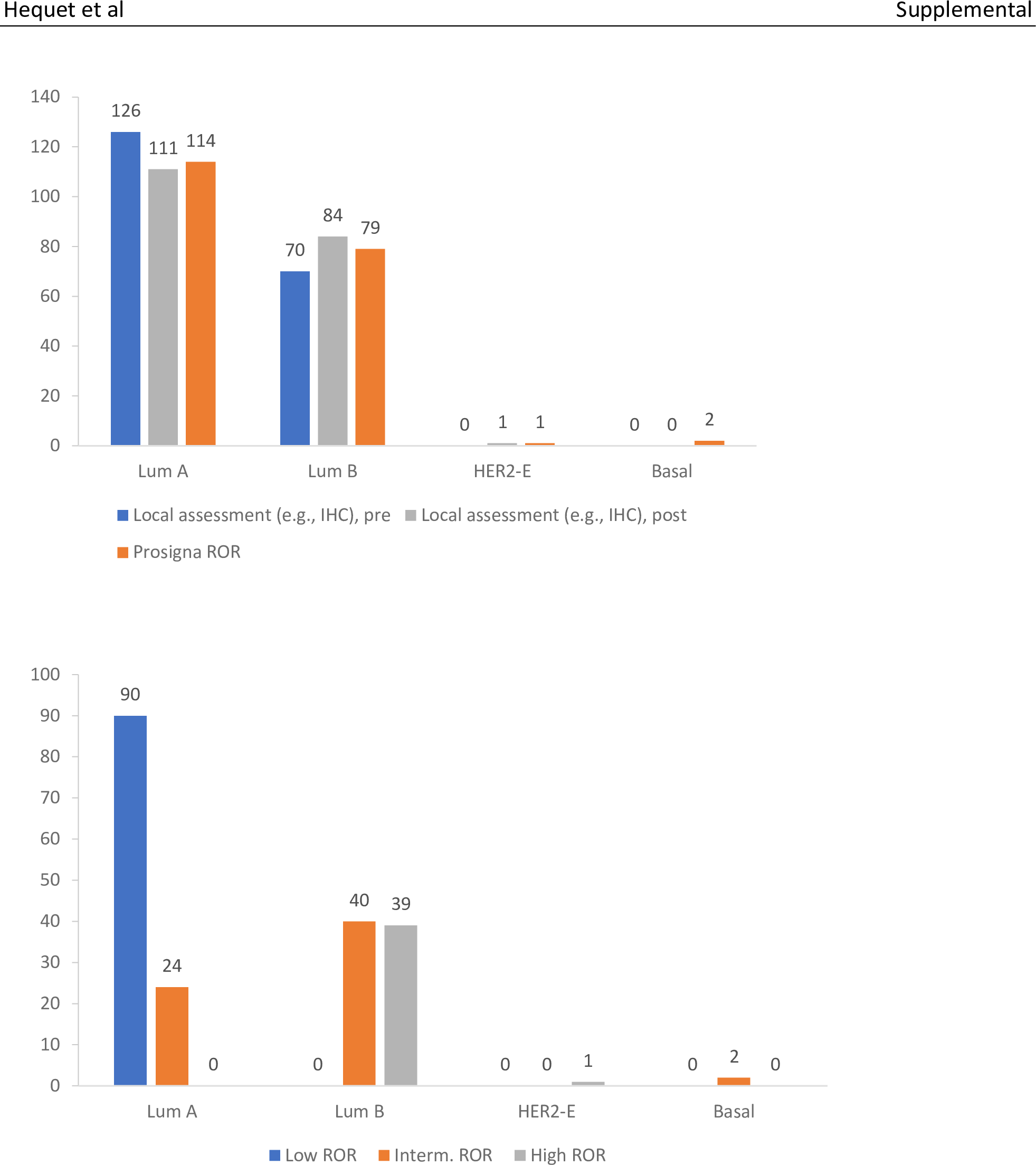

Supplement: S3 Fig — (TIF) [file pone.0185753.s003.tif]

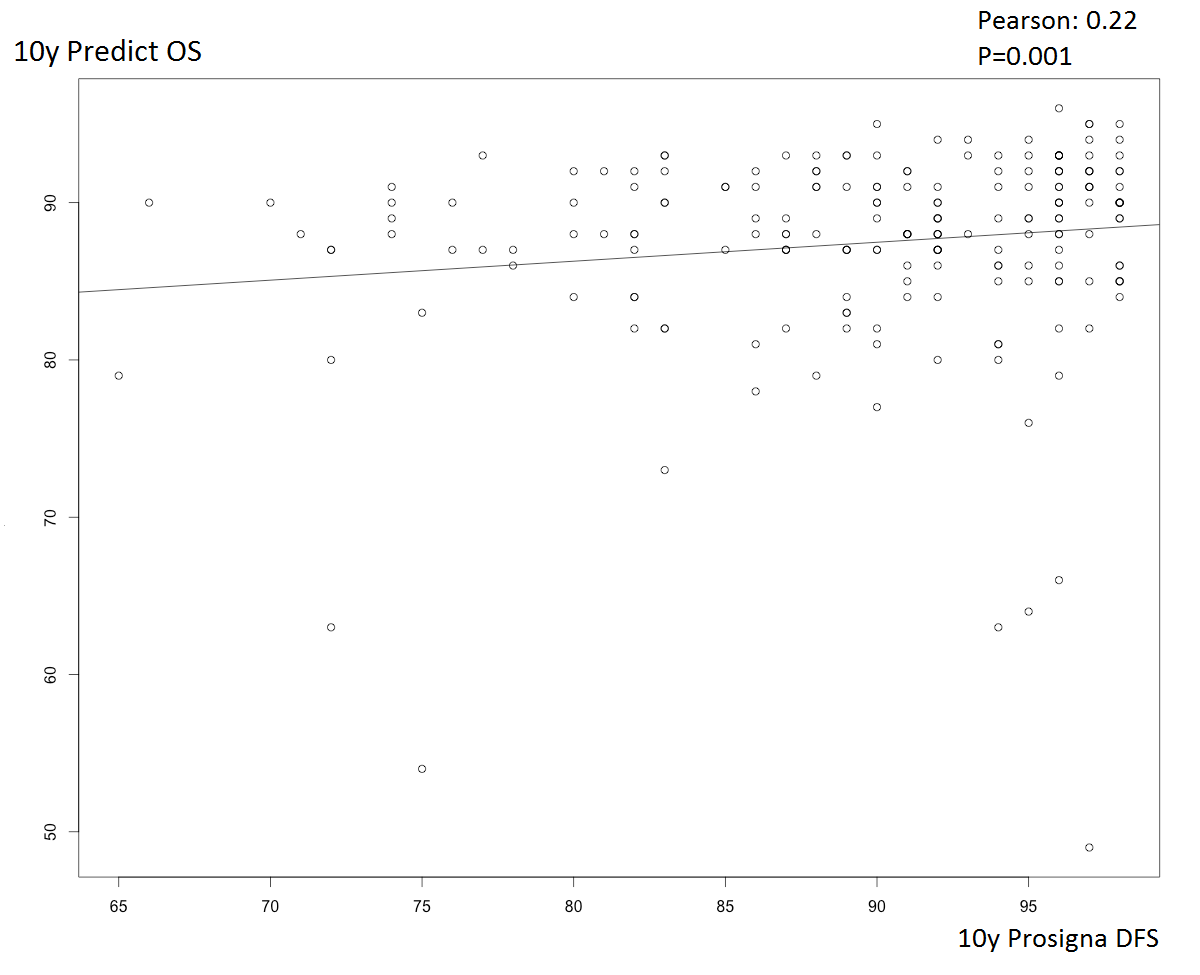

Supplement: S4 Fig — (TIF) [file pone.0185753.s004.tif]
