# Supplementary material for: Prospective, multicenter French study evaluating the clinical impact of the Breast Cancer Intrinsic Subtype-Prosigna® Test in the management of early-stage breast cancers
Source: PLoS One. 2017 Oct 18;12(10):e0185753. doi: 10.1371/journal.pone.0185753 (PMC5646764; doi:10.1371/journal.pone.0185753)
Supplement: S1 Table — (DOCX) [file pone.0185753.s005.docx]

**Supplemental Table 1 Changes in anxiety, decisional conflict, and functional status according to ROR group**

|  |  | **All patients** | | | |
| --- | --- | --- | --- | --- | --- |
|  | **Instrument** | **N** | **Mean** | **SD** | **p-value** |
|  | State Trait Anxiety Inventory |  |  |  |  |
|  | State-anxiety | 171 | -1.8 | 10.3 | **0.02** |
|  | Trait-anxiety | 169 | -0.8 | 6.5 | 0.115 |
|  | Decision Conflict Scale | 158 | -3.5 | 10.7 | **<0.001** |
|  | Informed | 164 | -7.7 | 17.8 | **<0.001** |
|  | Values clarity | 163 | -5.2 | 15.3 | **<0.001** |
|  | Support | 166 | -0.2 | 10.5 | 0.806 |
|  | Uncertainty | 166 | -3.6 | 17.2 | **0.008** |
|  | Effective decision | 165 | -1.8 | 17.9 | 0.207 |
|  | Functional Assessment | 151 | 0.9 | 9.5 | 0.264 |
|  | Physical well-being | 162 | 0.1 | 3.7 | 0.761 |
|  | Social/family well-being | 163 | 0.0 | 3.3 | 0.890 |
|  | Emotional well-being | 168 | 0.8 | 2.8 | **<0.001** |
|  | Functional well-being | 168 | -0.2 | 3.4 | 0.413 |
|  | Abbreviations: SD - standard deviation; max – maximum; min – minimum; Prosigna™ – Breast Cancer Intrinsic Subtyping Test; ROR – risk of recurrence. | | | | |

| **Instrument** | **Low ROR** | | | **Intermediate ROR** | | | **High ROR** | | | **P-value (ANOVA)** |
| --- | --- | --- | --- | --- | --- | --- | --- | --- | --- | --- |
| State Trait Anxiety Inventory | **Pre mean (SD)** | **Post mean (SD)** | **Mean Difference (SD)** | **Pre mean (SD)** | **Post mean (SD)** | **Mean Difference (SD)** | **Pre mean (SD)** | **Post mean (SD)** | **Mean Difference (SD)** |  |
| State-anxiety | 43.4 (11.4) | 39.0 (11.7) | -4.4 (8.1) | 43.1 (11.0) | 43.9 (13.0) | 0.9 (10.2) | 43.4 (13.4) | 43.6 (14.1) | 0.2 (14.2) | **0.005** |
| Trait-anxiety | 41.5 (10.3) | 40.6 (11.4) | -0.9 (5.9) | 40.6 (10.4) | 39.4 (11.3) | -1.3 (7.1) | 38.8 (12.1) | 39.6 (10.9) | 0.7 (6.9) | 0.423 |
| Decision Conflict Scale | 9.9 (10.0) | 5.8 (7.1) | -4.1 (9.3) | 9.3 (9.8) | 6.8 (8.7) | -2.5 (11.7) | 10.3 (13.7) | 6.3 (7.4) | -4.0 (12.3) | 0.697 |
| Informed | 14.5 (18.3) | 5.7 (9.4) | -8.8 (15.4) | 12.1 (17.0) | 4.8 (8.4) | -7.3 (18.4) | 11.0 (22.5) | 6.0 (9.3) | -5.1 (22.8) | 0.615 |
| Values clarity | 9.2 (12.4) | 4.4 (7.4) | -4.7 (9.3) | 12.2 (19.5) | 6.5 (11.4) | -5.7 (21.4) | 10.7 (14.1) | 5.4 (8.2) | -5.4 (15.1) | 0.942 |
| Support | 5.7 (9.7) | 4.8 (9.4) | -0.8 (10.0) | 3.9 (8.7) | 4.8 (10.0) | 0.9 (11.8) | 5.2 (9.6) | 4.6 (9.1) | -0.6 (9.7) | 0.633 |
| Uncertainty | 14.3 (15.4) | 11.1 (15.4) | -3.1 (19.6) | 12.7 (12.3) | 9.2 (10.9) | -3.5 (14.2) | 18.4 (22.8) | 13.5 (16.1) | -4.9 (16.0) | 0.896 |
| Effective decision | 9.2 (15.0) | 7.4 (13.9) | -1.8 (17.4) | 9.2 (15.0) | 9.4 (14.0) | 0.1 (19.6) | 11.5 (18.7) | 6.0 (10.2) | -5.5 (15.9) | 0.395 |
| Functional Assessment | 79.4 (13.0) | 80.3 (15.3) | 0.9 (8.2) | 78.7 (13.6) | 78.6 (15.3) | -0.1 (10.1) | 80.6 (14.1) | 83.0 (15.1) | 2.4 (11.5) | 0.559 |
| Physical well-being | 22.7 (4.3) | 22.9 (5.0) | 0.3 (3.6) | 22.7 (4.5) | 22.7 (5.0) | 0.0 (3.4) | 23.7 (4.2) | 23.5 (4.4) | -0.3 (4.5) | 0.780 |
| Social/family well-being | 21.4 (4.4) | 21.2 (4.2) | -0.1 (2.6) | 21.3 (4.3) | 20.7 (4.5) | -0.6 (3.4) | 20.8 (4.2) | 22.2 (4.3) | 1.3 (4.5) | **0.045** |
| Emotional well-being | 16.7 (4.2) | 17.5 (4.3) | 0.8 (2.5) | 17.0 (4.1) | 17.5 (4.1) | 0.5 (2.9) | 16.8 (4.0) | 17.9 (4.7) | 1.0 (3.2) | 0.669 |
| Functional well-being | 18.4 (4.1) | 18.3 (4.7) | -0.1 (3.1) | 19.1 (4.1) | 18.7 (4.9) | -0.4 (3.4) | 19.5 (4.7) | 19.2 (4.8) | -0.3 (4.3) | 0.894 |

Abbreviations: SD - standard deviation; max – maximum; min – minimum; Prosigna™ – Breast Cancer Intrinsic Subtyping Test; ROR – risk of recurrence.
