# Supplementary material for: Prospective, multicenter French study evaluating the clinical impact of the Breast Cancer Intrinsic Subtype-Prosigna® Test in the management of early-stage breast cancers
Source: PLoS One. 2017 Oct 18;12(10):e0185753. doi: 10.1371/journal.pone.0185753 (PMC5646764; doi:10.1371/journal.pone.0185753)
Supplement: S2 Table — (DOCX) [file pone.0185753.s006.docx]

**Supplemental Table 2. Anxiety, Decisional conflict, and functional status pre- and post-Prosigna, and at 6-month follow- up**

| **Instrument** | **Pre-Prosigna** | | **Post-Prosigna™** | | **6 months** | |
| --- | --- | --- | --- | --- | --- | --- |
|  | **n** | **Mean (SD)** | **n** | **Mean (SD)** | **n** | **Mean (SD)** |
| State Trait Anxiety Inventory |  |  |  |  |  |  |
| State-anxiety | 171 | 43.3 (11.6) | 171 | 41.5 (12.7) | 162 | 39.90 (12.28) |
| Trait-anxiety | 169 | 40.8 (10.6) | 169 | 40.0 (11.2) | 160 | 40.32 (11.73) |
| Decision Conflict Scale | 158 | 9.8 (10.6) | 158 | 6.2 (7.7) | Not Reported | |
| Informed | 164 | 13.1 (18.6) | 164 | 5.4 (9.1) |  |  |
| Values clarity | 163 | 10.5(15.4) | 163 | 5.3 (9.1) |  |  |
| Support | 166 | 5.0 (9.3) | 166 | 4.8 (9.5) |  |  |
| Uncertainty | 166 | 14.5 (16.0) | 166 | 10.9 (14.0) |  |  |
| Effective decision | 165 | 9.6 (15.6) | 165 | 7.8 (13.4) |  |  |
| Functional Assessment | 151 | 79.4 (13.3) | 151 | 80.2 (15.2) | 161 | 76.77 (15.77) |
| Physical well-being | 162 | 22.9 (4.4) | 162 | 22.9 (4.9) | 165 | 20.07 (5.21) |
| Social/family well-being | 163 | 21.2 (4.3) | 163 | 21.2 (4.3) | 165 | 20.71 (4.32) |
| Emotional well-being | 168 | 16.8 (4.1) | 168 | 17.5 (4.3) | 165 | 17.64 (4.54) |
| Functional well-being | 168 | 18.8 (4.2) | 168 | 18.6 (4.8) | 163 | 18.38 (5.11) |
