# Supplementary material for: Prospective, multicenter French study evaluating the clinical impact of the Breast Cancer Intrinsic Subtype-Prosigna® Test in the management of early-stage breast cancers
Source: PLoS One. 2017 Oct 18;12(10):e0185753. doi: 10.1371/journal.pone.0185753 (PMC5646764; doi:10.1371/journal.pone.0185753)
Supplement: S2 Appendix — dx.doi.org/10.17504/protocols.io.jrucm6w (DOC) [file pone.0185753.s008.doc]

**Prospective study evaluating the clinical impact of the Breast Cancer Intrinsic Subtype-Prosigna^TM^ Test (Assay) in the management of early-stage breast cancers.**

**Principal Investigator**

Prof. Roman Rouzier

Institut Curie

26 rue d’Ulm, 75005 Paris

[roman.rouzier@curie.fr](mailto:roman.rouzier@curie.fr)

| **Sponsor**  NanoString Technologies, Inc.  530 Fairview Ave N  Suite 2000  Seattle, WA 98109 USA |
| --- |
| **Data Management and Monitoring** |
| Dr. Delphine Héquet  Institut Curie, 26 rue d’Ulm, 75005 Paris  [delphine.hequet@curie.fr](mailto:delphine.hequet@curie.fr)  **Scientific co-coordinator**  John Hornberger, MD, MS  Cedar Associates, LLC  3715 Haven Ave, Suite 100  Menlo Park, CA 94025 USA  650-257-3315  **Central laboratory** |
| Plateforme de génomique (genomics platform), Institut Curie, David Gentien  26 rue d’Ulm, 75005 Paris, France  [david.gentien@curie.fr](mailto:david.gentien@curie.fr) |
| **Second laboratory** |
| Laboratoire d’anatomopathologie (Anatomic Pathology Laboratory), Centre Jean Perrin  58 Rue Montalembert, 63000 Clermont-Ferrand  [frederique.penault-llorca@cjp.fr](mailto:frederique.penault-llorca@cjp.fr) |

**STUDY PROTOCOL**

**Prospective study evaluating the clinical impact of the Breast Cancer Intrinsic Subtype-Prosigna^TM^ Test (Assay) in the management of early-stage breast cancers.**

**Agreement of the Sponsor**

_______________________________________ _____________________

Matthew J. Ellis, MD, PhD Date

In Representation of NanoString® Technologies, Inc.

**STUDY PROTOCOL**

**Prospective study evaluating the clinical impact of the Breast Cancer Intrinsic Subtype-Prosigna^TM^ Test (Assay) in the management of early-stage breast cancers.**

Signature of Agreement for Protocol I have read and approved the protocol and will carry out this study according to the rules described below

_______________________________________ _____________________

Prof. Roman Rouzier Date

Principal Investigator

Institut Curie

**STUDY PROTOCOL**

**Prospective study evaluating the clinical impact of the Breast Cancer Intrinsic Subtype-Prosigna^TM^ Test (Assay) in the management of early-stage breast cancers.**

**Signature of approval of the protocol**

I have read and approved the protocol and will carry out this study according to the rules described below

_______________________________________ _____________________

John Hornberger, MD, MS Date

Scientific Co-Coordinator

Cedar Associates, LLC

**TABLE OF CONTENTS**

**1. Statement of the question and objectives**

Statement of the question

Primary objective

Secondary objectives

**2. Study Population**

Inclusion criteria

Exclusion criteria

Recruitment and inclusion of patients

Training of investigators

**3. Methodology**

Data anonymization

Performing the Prosigna^TM^ genomic test (assay)

Gathering of data and modes of data circulation

Centers participating in the study

**4. Creation of a specimen bank**

**5. Data analysis**

Primary Endpoint

Seconday Endpoints

Method of analysis

Power analysis

**6. Monitoring of the study**

**7. Ethics committee and approval from regulatory authorities**

Informed consent

Patient confidentiality

Recommendations from the regulatory authorities

**7. Publications**

**8. Bibliography**

**9. Appendices**

Pre-Prosigna Patient CRF (Case Report Form)

Pre-Prosigna Physician CRF (Case Report Form)

Post-Prosigna Patient CRF (Case Report Form)

Post-Prosigna Physician CRF (Case Report Form)

Patient follow-up CRF (Case Report Form)

Physician follow-up CRF (Case Report Form)

Information letter and informed consent form

**1. Statement of the question and objectives**

**Statement of the question**

The tools of breast cancer diagnosis consist of the clinical examination, radiological examinations, and the anatomopathological analysis associated with immunohistochemistry. A classification of breast tumors into subtypes allows one to better evaluate the risk of recurrence and therefore to choose the optimal adjuvant therapy for the patients. For more than 10 years, research, clinical studies and reviews have permitted one to highlight the value of the classification into intrinsic subtypes of tumors based on gene expression to better define the prognosis and therefore adapt the adjuvant therapy (Gnant, Harbeck, and Thomssen 2011; Perou et al. 2000; Sorlie et al. 2001; Hornberger et al. 2012). The genomic tests currently available are Oncotype DX®, Endopredict®, and Mammaprint. The fundamental work carried out by Perou and Sorlie (Perou et al., 2000; Sorlie et al., 2001), permitted a better understanding of breast cancer. The pathology is considered in different molecular subtypes which influence the therapeutic response and the prognosis; this approach was notably adopted during the international conference of St. Gallen in 2011. Therefore, the therapeutic indications (hormone therapy, chemotherapy, Herceptin) are adapted to the different subtypes: Luminal A, Luminal B, HER2 positive and triple negative. The Prosigna™ test (assay) by NanoString® Technologies, Inc. (Seattle, WA), measures the level of expression of 50 genes from the tissue specimens fixed in formalin and embedded in paraffin and permits the classification of the tumors into subtypes and gives a score for the risk of recurrence at 10 years. The first clinical validation study of the Prosigna™ test (assay) on 1,017 specimens and comparing the results to Oncotype DX® has highlighted a more significant prognosis power of the Prosigna™ test (assay) with a lower number of patients classified as intermediate risk (Dowsett M et al. 2011). This tool in prognosis and in helping with the therapeutic decision will soon be used in common practice. It is therefore necessary to evaluate within a controlled clinical trial the impact of the test result on the therapeutic modifications on one hand and on the feelings of the physician and the patient on the other hand.

**The primary objective** of this study is to evaluate the impact of the result of the Prosigna^TM^ Test (assay) on the therapeutic decision of adjuvant therapy (chemotherapy and hormone therapy) in patients suffering from early-stage breast cancer. The following will be considered as therapeutic changes:

**The secondary objectives** will consist of

- The evaluation of the confidence in the therapeutic indications of the practitioners before and after the test (assay) results, and by subgroups of cancers

- The evaluation of the rate of secondary effects of chemotherapy,

- The evaluation of the emotional state of patients faced with the therapeutic decision, of their degree of anxiety and of their functional status before and after the Prosigna^TM^ test (assay) results

**2. Study Population**

All of the patients managed consecutively in the participating centers and meeting the inclusion criteria will be offered participation in the study. The patients who are incapable of signing an informed consent form will not be included in the study.

**Inclusion criteria**

- Invasive early-stage breast cancer (T1-T2, N0, pN0(i+), pN0 (micro-metastatic), M0), operated on immediately, with positive estrogen receptors, non-overexpressed HER2
  - The status of the hormonal receptors in the estrogen will be evaluated by immunohistochemistry. A fixation of more than 1% of the cells will be considered as positive.
  - The HER2 status will be evaluated by immunohistochemistry (IHC) and/or fluorescence in situ hybridization (FISH). (0 or 1+ or 2+ in IHC will be considered as negative in the absence of FISH).
- The patients must be post-menopausal. The "post-menopausal" status will be defined by:
  - Amenorrhea for more than 12 months, regardless of the age
  - Medical history of bilateral ovariectomy (oophorectomy) (surgery carried out, at the minimum, 4 weeks before the inclusion), regardless of the age
  - Radiological castration (cessation of ovarian function) with amenorrhea for more than 3 months, regardless of the age
  - Medical history of hysterectomy with hormonal biological doses in favor of menopause.
- Patient capable of giving her consent
- Patient eligible for therapy through adjuvant chemotherapy
- ECOG score of 0 or 1

**Exclusion criteria**

- Tumor size T3-T4
- Non-invasive breast cancer (CIS, Paget.)*
- Nodal status other than N0, pN0 (i+), or pN0 (mol+)
- Tumor that is estrogen-receptor negative or has overexpressed HER2
- Metastasis
- Patient incapable of giving her consent
- Patient incapable of filling out the questionnaires
- Contraindication to chemotherapy
- ECOG Score >1

**Recruitment and inclusion of patients**

The patients will be informed by the investigator of the terms of the study and of the legal provisions of a clinical research study. They will receive a written letter of information (presented as an appendix). The patients, at any time during the study, will be able to contact the investigator for additional information. The contact details of the investigators will be shown on the information letter. The patients will sign an informed consent form in duplicate giving their agreement for a 6 month follow-up and the use of their tissue specimen for future studies. The patients will be included in different centers, and no centers will be represented disproportionately. The informed consent form will be sent to the Institut Curie for review. In case of refusal of participation of a patient, the consultation date and the reasons for refusal will be documented. To complete the inclusion of a patient, the investigator will connect to a secure site using a personal login and password. The inclusion instructions will be available on the site.

The number of patients to be included is 200.

**Training of investigators**

The Institut Curie will coordinate the contacts with the investigators of each center and will provide the training of the investigators concerning the inclusions, the terms of the study and the informed consent forms.

NanoString Technologies will provide the training of the anatomic pathology laboratories of each center and will provide them with a written protocol explaining the preparation of the specimens necessary for the completion of the test (assay).

The tests (assays) will be carried out on the genomic platform of the Institut Curie in Paris and at the Centre Jean Perrin in Clermont-Ferrand. The members of these laboratories have already been trained in the performing of the Prosigna^TM^ tests (assays) using the NanoString nCounter technology. Internal controls will be organized regularly within the platform.

**3. Methodology**

This is a French multicenter prospective study.

**Data anonymization**

The information concerning the patients will be kept strictly confidential. Before data collection in the eCRF, the patients will be de-identified and an identification number will be assigned to them. The anonymization number (3 digits) will correspond to the participating center for the first digit and in the order of the consecutive inclusion of the patient of each center. The list of the identification numbers linked to the patient identities will be kept by the principal investigator of the center in the investigation file.

**Performing the Prosigna^TM^ genomic test (assay)**

The Prosigna^TM^ genomic test (assay) will be carried out on the surgical specimens of patients who have agreed to participate in the study, within the genomic platform of the Institut Curie. The test results will be expected within 7-10 days.

The anatomic pathology laboratory of each center trained on the study, once it has the informed consent form, will be in charge of the preparation of the specimen and for the delivery of the specimen to the genomic platform of the Institut Curie. The anonymization number provided during the inclusion by the investigator will be reported on the specimen. This specimen will be sent by courier (FedEx ® Clinical Pack) to the genomic platform of the Institut Curie where RNA extraction and the Prosigna^TM^ test (assay) will be carried out. The results will be transmitted to the investigator within a time period of 7-10 days in the form of a report containing the patient identification number in the study, the molecular subtype and the risk of recurrence at 10 years. These results will be available both on the secure server dedicated to the study, and linked to the patient using the patient identification number, and also transported by courier to the anatomic pathology laboratory of the center which is managing the patient.

**Gathering of data and modes of data circulation**

Once the consent form is signed and before the test results, the investigator and the patient will complete the Case Report Form (CRF), pre-Prosigna. The physician will provide the information concerning the patient and disease characteristics, and of the adjuvant therapy planned. At the inclusion visit (signing of the informed consent form), the patients will fill out pre-Prosigna questionnaires concerning their state of anxiety, the difficulties felt in face of the therapy and the quality of life. The CRFs of the study will be presented in an electronic form (eCRF). Access to the eCRF server will be secured by personal access codes provided to each investigator by the principal investigator/data manager. Each investigator will have access to the anonymized data of the patients that he or she will have included in his or her center. Access to all of the data will only be accessible to the principal investigator/data manager. This data will be stored on a secure server.

The anonymized anatomic pathology report, as well as the results from the Prosigna^TM^ test (assay) will be collected and stored with the eCRFs.

Once the test (assay) results are obtained, the investigator and the patient will complete the post-Prosigna CRF. Information regarding the adjuvant therapy decision will be collected again, as well as the patient's state of anxiety and her difficulties felt, faced with the therapy.

During a 6 month inclusion follow-up visit, the patients will fill out the follow-up CRF in which information regarding their degree of anxiety, their possible difficulties felt facing the therapy and their quality of life will be collected. The physicians will also fill out a 6 month CRF concerning their opinion on the usefulness of the test (assay), their confidence in the test results and the patients' medical follow-up elements.

The various CRFs are shown as appendices.

**Centers participating in the study**

Participating hospitals: Institut Curie (Paris and St-Cloud sites), Hôpital Tenon-AP-HP-Paris, Centre hospitalier Poissy-St Germain, Centre hospitalier René Dubos-Pontoise, Clinique des Peuplier-Paris, Institut Gustave Roussy-Villejuif, Centre Jean Perrin-Clermont-Ferrand, and Private hospital Clairval-Marseille.

**4. Creation of a specimen bank**

During this study, a breast cancer tissue bank will be formed. For each patient participating in the study, a paraffin-embedded tumor section will be sent to the Institut Curie to carry out the Prosigna^TM^ test (assay) and may be used for future projects of translational research. The collection of tumor specimens in this study will be carried out according to the recommendations published in the “Recommendations for collection and handling of specimens from group breast cancer clinical trials” .

**5. Data analysis**

**Primary Endpoint**

Evaluation of the proportion of patients for which the therapy was modified following the results of the Prosigna^TM^ Test (assay).

**Secondary Endpoints**

• Proportion of patients for which the therapy was modified, stratified by cancer recurrence risk group (low, intermediate, high)

• Proportion of patients for which the therapy was modified, for the sub-group of patients who have a recurrence risk close to the limits between groups (+/- 5% of the limits).

• Confidence of the practitioners in the therapeutic recommendations before and after the results from the Prosigna^TM^ Test (assay) for each sub-group (low, intermediate and high risk of recurrence).

• Modifications of the patients' difficulties linked to the choice of therapy, of the degree of anxiety and of the functional status of the patients before and after the Prosigna^TM^ Test (assay), stratified by the sub-groups of recurrence risk.

• Rate and severity of the secondary effects of the therapy, according to the administration or non-administration of chemotherapy.

• Agreement of the classification into molecular subtypes between the Prosigna^TM^ Test (assay) and the immunohistochemistry.

• Retrospective research on the tissue specimens collected.

**Method of analysis**

The clinical and demographic characteristics of the inclusion population will be described by mean, median, standard deviation, range and frequencies. The proportion of patients for whom the choice of therapy from the physician has changed before and after the Prosigna^TM^ test (assay) results will be calculated with a confidence interval of 95%. The confidence changes of the practitioners in the therapies carried out before and after the test (assay) results will be analyzed by calculating the mean (CI 95%).

**Power analysis**

In order to obtain a confidence interval of 95%, we will include 200 patients in total (all centers combined).

Table 1 Calculation of the specimen size according to the confidence interval

| Confidence interval | Proportion (P) | Lower limit | Distance from P to lower limit | | Sample size (N) |
| --- | --- | --- | --- | --- | --- |
| 0.95 | 0.25 | 0.20 | 0.05 | 200 | |
| 0.95 | 0.25 | 0.19 | 0.06 | 137 | |
| 0.95 | 0.25 | 0.18 | 0.07 | 100 | |
| 0.95 | 0.25 | 0.17 | 0.08 | 76 | |
| *References: Fleiss, J. L., Levin, B., Paik, M.C. 2003. Statistical Methods for Rates and Proportions. Third Edition.*  *John Wiley & Sons. New York. Newcombe, R. G. 1998. 'Two-Sided Confidence Intervals for the Single Proportion:*  *Comparison of Seven Methods.' Statistics in Medicine, 17, pp. 857-872.* | | | | | |

Figure 1 Sample size depending on the confidence interval


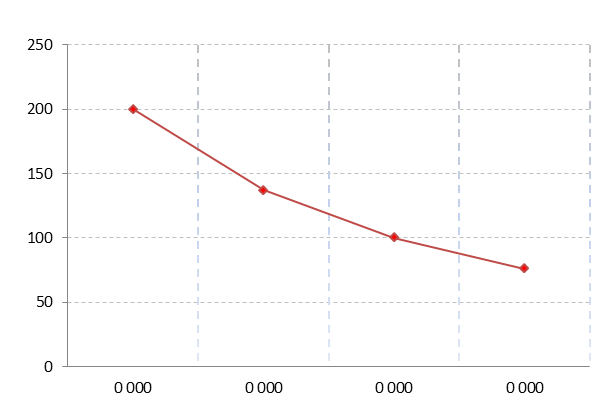


**Sample size (N)**

**Confidence Interval**

**6. Monitoring of the study**

In order to assure the proper conduct of the study, each site will receive 3 monitoring visits:

**First visit**

The first visit will take place in each center after inclusion of the first 5 patients. During this visit, a reminder of the study procedures will be carried out with the center investigators. The following data will be verified for each participant:

- Informed consent form actually signed
- Inclusion and exclusion criteria
- Pre-Prosigna patient CRF (correctly filled out, actually prior to the test)
- Pre-Prosigna physician CRF (correctly filled out, actually prior to the test)

**Second visit**

The second visit will take place in each center after inclusion of the last patient. The following data will be verified for each participant:

- Post-Prosigna patient CRF (correctly filled out, actually after the test)
- Post-Prosigna physician CRF (correctly filled out, actually after the test)

**Third visit**

The third visit will take place in each center after the 6 month follow-up of the last patient included. The following data will be verified for each participant:

- Patient follow-up Prosigna CRF (correctly filled out, actually at 6 months)
- Physician follow-up Prosigna CRF (correctly filled out, actually at 6 months)

Any deviation from the initial protocol will be documented and submitted to the principal investigator/data manager after each visit.

Additional monitoring will be carried out if necessary.

**7. Ethics committee and approval from regulatory authorities**

**Informed consent**

An informed consent form will be given to all of the patients meeting the inclusion criteria and consecutively managed in the centers participating in the study, after oral information delivered by the investigators of each center. The patients agreeing to participate will sign an informed consent form shown in the appendix of this protocol. The patients will be invited to contact the investigators throughout the study, and the contact details of the investigators will be provided in the informed consent form. The investigators of each center may contact the principal investigator/data manager at any time during the study.

**Patient confidentiality**

All of the information concerning the patients will be kept strictly confidential. From the inclusion in the study, the data will be anonymized. The identification number will be used to report the patient and physician CRFs as well as the test (assay) results.

**Recommendations from the regulatory authorities**

The ethics committee of the Institut Curie has approved this study.

The Comité de Protection des Personnes de Paris V (Paris V Institutional Review Board (IRB)), has rendered a favorable opinion dated May 2, 2014. The study was registered under number 14952.

The opinion of the Comité Consultatif sur le Traitement de l'Information en matière de Recherche dans le domaine de la Santé (CCTIRS) (French Advisory Committee on Research Information Processing in the field of Health) was rendered on ____________ and has issued a _________ opinion.

Authorization from the Commission Nationale de l’Informatique et des Libertés (CNIL) (French National Commission for Data Protection and Liberties) was given on ___________.

Authorization from the Agence Nationale de Sécurité du Médicament et des produits de santé (ANSM) (French National Agency for Medicines and Health Products Safety) was given on _______________.

**7. Publications**

Any publication of the study results must require final approval from the principal investigator. The principal investigator will provide the last proofreading of the publications and will assure the respect of the patients' rights and the anonymity of the data. The list of the authors of the publications must be approved by the investigators of the study. The names and positions of the authors of the study will be decided according to the active participation in the design and the setting up of the study, the writing of the publication, and the statistical analysis. The number of patients recruited by the center will also be taken into consideration. In case of limitation of the number of authors for a review, the most active investigators and collaborators will be considered as authors.

**8. Bibliography**

- Dowsett M, Lopez-Knowles E, Sidhu K, et al. Comparison of PAM50 Risk of Recurrence(ROR) score with OncotypeDx and IHC4 for predicting residual risk of RFS and Distant-(D)RFS after endocrine therapy: a TransATAC Study. Presented at the CTRC-AACR San Antonio Breast Cancer Symposium; December 6-10, 2011; San Antonio, Texas. Abstract S4-5.
- Les cancers en France en 2013, Institut National du Cancer (The cancers in France in 2013, National Institute of Cancer) from http://www.e- cancer.fr/publications/69-epidemiologie/758-les-cancers-en-france-edition-2013l
- Gnant, M., N. Harbeck, and C. Thomssen. 2011. "St. Gallen 2011: Summary of the Consensus Discussion." Breast Care (Basel) no. 6 (2):136-141. doi: 10.1159/000328054.
- Hornberger, J., M. D. Alvarado, C. Rebecca, H. R. Gutierrez, T. M. Yu, and W. J. Gradishar. 2012. "Clinical Validity/Utility, Change in Practice Patterns, and Economic Implications of Risk Stratifiers to Predict Outcomes for Early-Stage Breast Cancer: A Systematic Review." J Natl Cancer Inst. doi: 10.1093/jnci/djs261.
- Leyland-Jones, B. R., C. B. Ambrosone, J. Bartlett, M. J. Ellis, R. A. Enos, A. Raji, M. R. Pins, J. A. Zujewski, S. M. Hewitt, J. F. Forbes, M. Abramovitz, S. Braga, F. Cardoso, N. Harbeck, C. Denkert, and S. D. Jewell. 2008. "Recommendations for collection and handling of specimens from group breast cancer clinical trials." J Clin Oncol no. 26 (34):5638-44. doi: 10.1200/JCO.2007.15.1712.
- Perou, C. M., T. Sorlie, M. B. Eisen, M. van de Rijn, S. S. Jeffrey, C. A. Rees, J. R. Pollack, D. T. Ross, H. Johnsen, L. A. Akslen, O. Fluge, A. Pergamenschikov, C. Williams, S. X. Zhu, P. E. Lonning, A. L. Borresen-Dale, P. O. Brown, and D. Botstein. 2000. "Molecular portraits of human breast tumours." Nature no. 406 (6797):747-52. doi: 10.1038/35021093.
- Siegel, R., E. Ward, O. Brawley, and A. Jemal. 2011. "Cancer statistics, 2011: the impact of eliminating socioeconomic and racial disparities on premature cancer deaths." CA Cancer J Clin no. 61 (4):212-36. doi: 10.3322/caac.20121.
- Sorlie, T., C. M. Perou, R. Tibshirani, T. Aas, S. Geisler, H. Johnsen, T. Hastie, M. B. Eisen, M. van de Rijn, S. S. Jeffrey, T. Thorsen, H. Quist, J. C. Matese, P. O. Brown, D. Botstein, P. E. Lonning, and A. L. Borresen-Dale. 2001. "Gene expression patterns of breast carcinomas distinguish tumor subclasses with clinical implications." Proc Natl Acad Sci U S A no. 98 (19):10869-74. doi: 10.1073/pnas.191367098.
- Viale, G., M. M. Regan, P. Dell'Orto, M. G. Mastropasqua, E. Maiorano, B. B. Rasmussen, G. MacGrogan, J. F. Forbes, R. J. Paridaens, M. Colleoni, I. Lang, B. Thurlimann, H.
- Mouridsen, L. Mauriac, R. D. Gelber, K. N. Price, A. Goldhirsch, B. A. Gusterson, and A. S. Coates. 2011. "Which patients benefit most from adjuvant aromatase inhibitors? Results using a composite measure of prognostic risk in the BIG 1-98 randomized trial." Ann Oncol no. 22 (10):2201-7. doi: 10.1093/annonc/mdq738.

**9. Appendices**

**Pre-Prosigna Patient CRF (Case Report Form)**

**Pre-Prosigna Physician CRF (Case Report Form)**

**Post-Prosigna Patient CRF (Case Report Form)**

**Post-Prosigna Physician CRF (Case Report Form)**

**Patient follow-up CRF (Case Report Form)**

**Physician follow-up CRF (Case Report Form)**

**Information letter and informed consent form**
